# Mathematical analysis identifies the optimal treatment strategy for epidermal growth factor receptor-mutated non-small cell lung cancer

**DOI:** 10.3389/fonc.2023.1137966

**Published:** 2023-09-28

**Authors:** Qian Yu, Susumu S. Kobayashi, Hiroshi Haeno

**Affiliations:** ^1^ Department of Computational Biology and Medical Sciences, Graduate School of Frontier Science, The University of Tokyo, Kashiwa, Japan; ^2^ Department of Medicine, Beth Israel Deaconess Medical Center, Harvard Medical School, Boston, MA, United States; ^3^ Research Institute for Biomedical Sciences, Tokyo University of Science, Noda, Japan

**Keywords:** computational modeling, drug resistance, cancer evolution, lung cancer, optimal treatment strategy

## Abstract

**Introduction:**

In Asians, more than half of non-small cell lung cancers (NSCLC) are induced by epidermal growth factor receptor (EGFR) mutations. Although patients carrying EGFR driver mutations display a good initial response to EGFR-Tyrosine Kinase Inhibitors (EGFR-TKIs), additional mutations provoke drug resistance. Hence, predicting tumor dynamics before treatment initiation and formulating a reasonable treatment schedule is an urgent challenge.

**Methods:**

To overcome this problem, we constructed a mathematical model based on clinical observations and investigated the optimal schedules for EGFR-TKI therapy.

**Results:**

Based on published data on cell growth rates under different drugs, we found that using osimertinib that are efficient for secondary resistant cells as the first-line drug is beneficial in monotherapy, which is consistent with published clinical statistical data. Moreover, we identified the existence of a suitable drug-switching time; that is, changing drugs too early or too late was not helpful. Furthermore, we demonstrate that osimertinib combined with erlotinib or gefitinib as first-line treatment, has the potential for clinical application. Finally, we examined the relationship between the initial ratio of resistant cells and final cell number under different treatment conditions, and summarized it into a therapy suggestion map. By performing parameter sensitivity analysis, we identified the condition where　osimertinib-first therapy was recommended as the optimal treatment option.

**Discussion:**

This study for the first time theoretically showed the optimal treatment strategies based on the known information in NSCLC. Our framework can be applied to other types of cancer in the future.

## Introduction

Among all the cancer types, lung cancer causes the highest number of cancer-related deaths. 26% of cancer-related deaths in males and 25% in females are induced by lung cancer (1). In Asians, 53% of non-small cell lung cancer (NSCLC) progression is induced by epidermal growth factor receptor (EGFR) mutations, such as the L858R mutation, exon 19 deletion, and exon 20 insertion (2). Besides, EGFR has been recognized as an oncogenic driver of NSCLC, making it increasingly important in the era of precision medicine for lung cancer (3).

EGFR belongs to the receptor tyrosine kinase (RTK) family. EGFR is activated by various ligands in the extracellular environment and transmits cellular responses to mediate many cellular activities, including cell proliferation, survival, growth, and development. It is expressed in many organs, with its abnormal expression associated with a variety of cancers. EGFR has an extracellular ligand-binding domain, hydrophobic transmembrane domain, and cytoplasmic tyrosine kinase domain. The driver mutations in EGFR associated with cancers are concentrated in the tyrosine kinase domain, forming exons 18–21 (4–7). More than 200 types of EGFR mutations have been identified, but the most common types are exon-19 deletion and the L858R mutation in exon 21 (8, 9). Approximately 44% of EGFR-mutated patients harbor exon-19 deletion, and 31% have the L858R mutation (10).

Although EGFR was first identified in 1977, EGFR-targeted antitumor drugs were first reported in 1994 (11). After the first report of EGFR-targeted therapy, first-generation EGFR-Tyrosine Kinase Inhibitors (EGFR-TKIs) were not approved until 2004 (12). Subsequently, the second-generation EGFR-TKI, afatinib, was approved in 2014. First- and second-generation EGFR-TKIs are effective in most cases of lung cancer harboring EGFR driver mutations (13–16). However, acquiring mutations, such as the T790M mutation, causes drug resistance and induces treatment failure (17, 18). In 2015, the third-generation EGFR-TKI (osimertinib), which inhibits both driver mutations and the T790M mutation, was approved as a second-line drug for patients with EGFR mutations (19–22). Although osimertinib is clinically effective, the emergence of additional mutations, such as the C797S mutation, induces resistance to osimertinib (23–25). Clinical observations suggest that optimized treatment schedules can help patients achieve better therapeutic effects (26–28). Thus, predicting resistance evolution and making reasonable treatment schedules in advance are necessary to delay the appearance of drug resistance and prolong the time of recurrence. However, even with knowledge of medical and genetic information in the early stage, such as tumor size and the proportion of different genotypes, it is still difficult to simulate the future development of tumors using traditional biological techniques alone.

Mathematical modeling is an approach for simulating realistic problems using mathematics and computational algorithms. This can offer a better understanding of how tumors evolve during treatment, which can be visualized *in vivo*. Thus, it can help us predict tumor dynamics under certain treatment schedules, compare different treatments, and even suggest optimal treatment strategies. Many studies have demonstrated the capability of mathematical modeling in cancer-related research (29–33). For example, Diaz Jr. et al. developed a mathematical model of cell kinetics during chemotherapy to predict the emergence of resistant genotypes in colorectal cancer (30). Castagnino et al. established a mathematical model of a genetic network to identify novel molecular targets for the treatment of colorectal cancer (34). In this way, we decided to employ mathematical modeling to predict tumor evolution and direct reasonable treatment schedules for lung cancer patients harboring EGFR mutations.

In this study, we establish a novel mathematical model of lung cancer evolution under EGFR-targeted therapy based on clinical observations. Parameter values in the model are estimated from published literature, and the results are confirmed using clinical observations. Moreover, we examine the relationship between the timing of switching drugs and the final number of cells in the tumor. Furthermore, we compare the combinatorial use of EGFR-TKIs to their sequential use to test their potential for clinical application. Finally, we investigate how intratumoral heterogeneity at the initial time of therapy affects treatment outcomes. The simulation results are comprehensively tested by parameter sensitivity analysis in order to identify the condition where each treatment strategy becomes the best option. Our framework is expected to be capable of suggesting reasonable individualized medicine for EGFR-mutated NSCLC.

## Materials and methods

### Mathematical model

**Figure 1:**
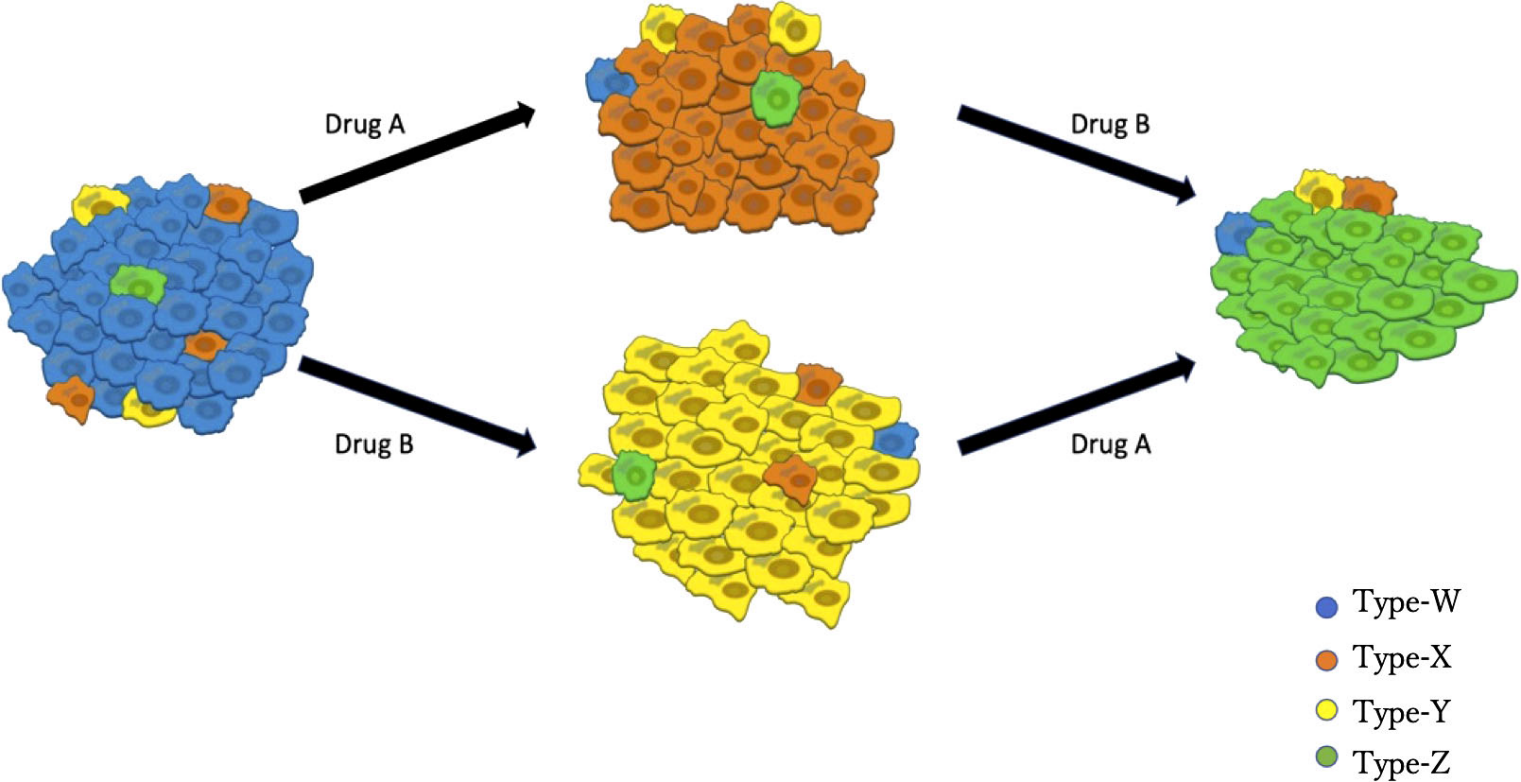
Illustration of the Model. Blue cells represent EGFR-TKI sensitive genotypes (for example, EGFR^L858R^ and EGFR^del-19^), orange cells represent osimertinib sensitive genotypes (such as EGFR^L858R-T790M^ or EGFR^del-19-T790M^), yellow cells represent osimertinib-only resistant genotypes (for instance, EGFR^L858R-C797S^ or EGFR^del-19-C797S^), green cells represent all EGFR-TKI resistant genotypes (like EGFR^L858R-T790M-C797S^ and EGFR^del-19-T790M-C797S^). DrugA involved the first- and second-generation EGFR-TKIs (erlotinib, gefitinib, and afatinib), and DrugB is osimertinib.

Based on clinical observations (35, 36), we established a mathematical model that describes the dynamics of the four types of EGFR-mutated cells under two types of EGFR-TKIs (**Figure 1**). There are two different types of EGFR-TKIs in the model: one is “DrugA,” representing the first- or second-generation EGFR-TKIs named gefitinib, erlotinib or afatinib; and the other is “DrugB,” representing osimertinib. Four cancer cell types are denoted by “Type-W,” “Type-X,” “Type-Y,” and “Type-Z”. Type-W is sensitive to both DrugA and DrugB, indicating cancer cells with driver EGFR mutations, such as L858R mutations or exon-19 deletion. Type-X cells are resistant to DrugA but sensitive to DrugB, indicating cells with T790M mutations. Type-Y is sensitive to DrugA but resistant to DrugB, indicating cells with C797S mutations. Type-Z is resistant to both DrugA and DrugB. Summarizing the relationship between drugs and cells, under DrugA treatment, Type-W and Type-Y will decrease, but Type-X and Type-Z will increase, whereas under DrugB treatment, Type-W and Type-X will decrease, but Type-Y and Type-Z will increase. According to published clinical studies (37–39), when DrugA was administered as first-line treatment, Type-X eventually became dominant in the tumor. After switching from DrugA to DrugB, the frequency of Type-X decreased, and only Type-Z continued to grow and dominate the tumor. However, when using DrugB as the first-line treatment, Type-Y will replace Type-W as the major population. After switching from DrugB to DrugA, only Type-Z became the donor population in the tumor.

In this mathematical model, we assumed that each cell type itself increases in number by cell division and mutates into a resistant type at a low mutation rate. We did not consider back mutations that were resistant to the sensitive cells. Moreover, according to the purpose of this study, we only focused on mutations related to drug resistance and assumed that other mutations are neutral and do not affect the growth kinetics. Then the dynamics of Type-W, Type-X, Type-Y, and Type-Z are given by Eqs. (1)


(1.1) $$\frac{dw}{dt}=aw$$

(1.2) $$\frac{dx}{dt}=gw+bx$$

(1.3) $$\frac{dy}{dt}=hw+cy$$

(1.4) $$\frac{dz}{dt}=kw+px+qy+fz$$

Here, the variables *w*, *x*, *y*, and *z* represent the cell numbers of Type-W, -X, -Y, and -Z, respectively. Parameters *a*, *b*, *c*, and *f* are the growth rates of Type-W, Type-X, Type-Y, and Type-Z, respectively, and *g*, *h*, *k*, *p*, and *q* are the mutation rates from type-W to Type-X, Type-W to Type-Y, Type-W to Type-Z, Type-X to Type-Z, and Type-Y to Type-Z, respectively. Because no other cell type can mutate into Type-W, the number of Type-W cells is affected by its kinetics only. However, Type-W will mutate into Type-X, Type-Y, and Type-Z.

### Solution of equations

The Eqs. (1) can be solved and given by Eqs. (2)


(2.1) $$w(t)=W_{0}{\text{e}}^{at}$$

(2.2) $$x(t)=X_{0}{\text{e}}^{bt}+\frac{g}{a-b}W_{0}{\text{e}}^{(at-bt){\text{e}}^{bt}}$$

(2.3) $$y(t)=Y_{0}{\text{e}}^{ct}+\frac{h}{a-c}W_{0}{\text{e}}^{(at-ct){\text{e}}^{ct}}$$

(2.4) $$\begin{array}{l} z(t)=Z_{0}{\text{e}}^{ft}-\frac{{\text{e}}^{ft}}{ab+ac-bc-a^{2}}[\frac{W_{0}}{a-f}{\text{e}}^{\left(at-ft\right)}(a^{2}k-abk-ack+bck+agp+\\ ahq-cgp-bhq)\\ +\frac{X_{0}}{b-f}{\text{e}}^{\left(bt-ft\right)}(a^{2}p-abp-acp+bcp)\\ +\frac{Y_{0}}{c-f}{\text{e}}^{\left(ct-ft\right)}(a^{2}q-abq-acq+bcq)] \end{array}$$

The equations describe the cell number of each type over time (*t*) during therapy. *W*_0_, *X*_0_, *Y*_0_, and *Z*_0_ represent the initial cell numbers of Type-W, -X, -Y, and Type-Z in the tumor. Please refer to **Table 1** for the meaning of each letter in the model.

**Table 1:** Parameter notation in the mathematical model.

Cell Type	Cell Number	Initial Cell Number	Growth Rate	Mutation Rate
Type-W	*w*	*W*_0_	*a*	/
Type-X	*x*	*X*_0_	*b*	g
Type-Y	*y*	*Y*_0_	*c*	h
Type-Z	*z*	*Z*_0_	*f*	*k* (from W), *p* (from X), *q* (from Y)

### Parameter evaluation

The parameter values were obtained from the published data (**Table 2**) (40–42). Since we obtained growth parameters under erlotinib and osimertinib treatments, we regarded these drugs as representative of DrugA and DrugB, respectively. Since Starrett et al. (41) reported that first-line therapy with erlotinib and osimertinib delayed the emergence of secondary mutations in untreated EGFR-mutated NSCLC, thus, for combination therapy, we defined a combinatorial regimen of erlotinib plus osimertinib as DrugC. Based on genome-editing cell line experiments (40), we adopted the growth rate of EGFR-L858R mutated cells for Type-W as -0.17 [/day] under DrugA (*a_A_*) and -0.32 [/day] under DrugB (*a_B_*). Note that the subscript of each growth rate represents the condition of drugs, *i.e.*, *a_A_* represents the growth rate of Type-W under DrugA. From Starrett et al. (41), we adopted the growth rate of EGFR-L858R/C797S mutated cells for Type-Y as -0.13 [/day] under DrugA (*c_A_*) and 0.024 [/day] under DrugB (*c_B_*). The growth rate of EGFR-L858R/T790M/C797S mutated cells for Type-Z is 0.022 [/day] under DrugB (*f_B_*), and the growth rate of EGFR-L858R/C797S mutated cells for Type-Y is -0.0335 [/day] under DrugC (*c_C_*). According to Gunnarsson et al. (42), we set all the mutation rate as 10^-7^ [/day] (*g, h, k, p* and *q*).

**Table 2:** Parameter values with different therapies.

Parameters Drug	Growth Rate (/day)				Mutation Rate (/day)				
	a (L858R)	b (L858R/T790M)	c (L858R/C797S)	f (L858R/T790M/C797S)	g (W→X)	h (W→Y)	k (W→Z)	p (X→Z)	q (Y→Z)
A(erlotinib)	-0.17	0.045	-0.13	0.022	10^-7^	10^-7^	10^-7^	10^-7^	10^-7^
B(osimertinib)	-0.32	-0.15	0.024	0.022	10^-7^	10^-7^	10^-7^	10^-7^	10^-7^
C(e+0)	-0.064	-0.0335	-0.0335	0.022	10^-7^	10^-7^	10^-7^	10^-7^	10^-7^

Based on the adopted parameters, we assumed all the other parameter values. Because the growth rate of Type-Y under DrugC is approximately 26% of that under DrugA (-0.0335/-0.13), we calculated the growth rate of Type-W in DrugC as 26% of that under DrugA, which is -0.064 [/day] (*a_C_*). We assume the growth rate of Type-X under DrugC is same as Type-Y, which is -0.0335 [/day] (*b_C_*). Moreover, we assumed the growth rate of Type-X under DrugB as -0.15 [/day] (*b_B_*), which is smaller than that of Type-Y under DrugA (*c_A_*) based on clinical observation (20, 21) where the first-line treatment by DrugB showed better prognosis than that by DrugA. Based on the same reason, we assumed the growth rate of Type-X cell under DrugA as 0.045 [/day] (*b_A_*). Finally, since Type-Z is resistant to both DrugA and DrugB, we assume its growth rates under DrugA and DrugC are same as that under DrugB effect, which is -0.022 [/day] (*f_A_* and *f_C_*).

As for the initial condition of simulations, the initial total cell number of the tumor is set to be 10^9^, and the standard initial cell number of Type-X (*X*_0_), Type-Y (*Y*_0_), Type-Z (*Z*_0_), and Type-W (*W*_0_), is 10^4^, 10^4^, 10, and the rest component, respectively. The initial total cell number is set to be 10^9^ because the diameter of a tumor at this point is about 1cm and a detectable size clinically. About the drug switching time in monotherapy, we set day-307 (sta=307) under DrugA-first therapy and day-567 (stb=567) under DrugB-first therapy based on clinical statistic data of the median Progression-Free Survival (mPFS) (20). The whole treatment time is assumed to be 1000 days (T=1000) in our simulation because 1000 days is long enough to compare the treatment options.

### Computational simulation

We used Python (version 3.8.8) to simulate our model. We did time course simulation of different therapies for checking whether our model can express the progression of tumor as clinical observation. Then, we simulated the relationship between drug switching time and final cell number to theoretically figured out the possible affects that could influence therapy effects. Moreover, we simulated how parameters affected final cell number in different therapies. All the codes for simulations can be found in our GitHub open repository: https://github.com/yuqianxigua/EGFR-TKIs-therapy.

## Results

### Tumor evolution under different treatments

**Figure 2:**
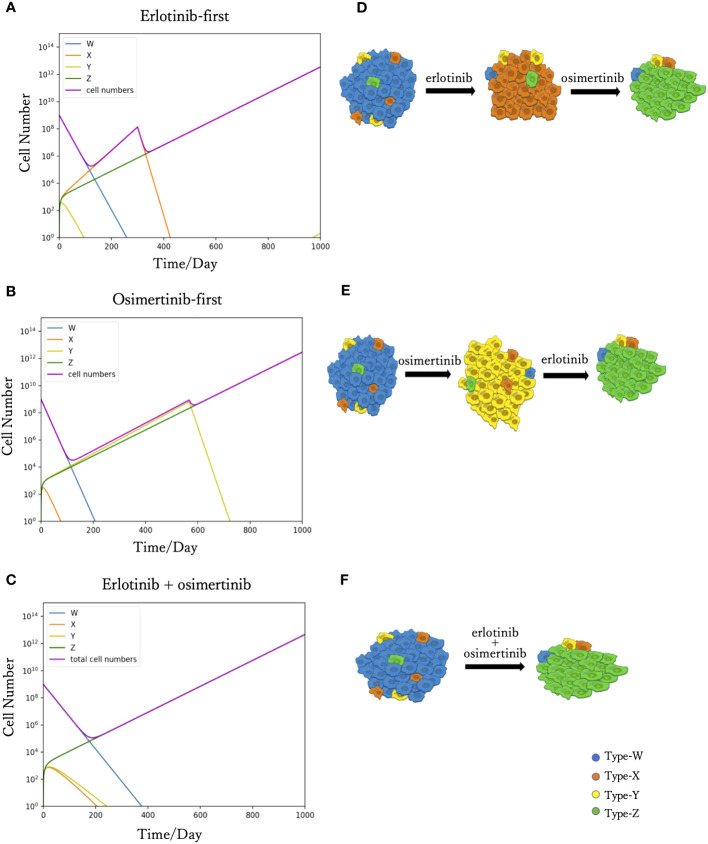
Time course simulation results of monotherapy and combination therapy. The results of the simulations are depicted in **(A–C)**. The x-axis is time, and the y-axis is the cell number. The blue, orange, yellow and green curves represent the dynamics of Type-W, -X, -Y, and -Z, respectively. The purple curve represents the total cell number. The expected tumor progression tendencies are depicted in **(D, E, F)**. The blue, orange, yellow, and green cells are Type-W, -X, -Y, and -Z, respectively. In the simulation of erlotinib-first **(A)**, the main population changed from Type-W to Type-X for a while. After changing erlotinib to osimertinib at day 307, Type-X decreased, and Type-Z became the dominant population in the end. This simulation result represents the tumor evolution tendency shown in **(D)**. The simulation product of osimertinib-first is shown in **(B)**. The tumor response to osimertinib increased in the beginning, but as Type-Y became the main population, osimertinib-resistance appeared. After the change to erlotinib at day 567, Type-Y decreased, and the tumor response to treatment increased again. However, Type-Z became the central population causing drug resistance. This simulation result represents the tumor evolution tendency shown in **(E)**. In the combination therapy **(C)**, the main population changed from Type-W to Type-Z. This result represents the tumor evolution tendency shown in **(F)**.

We simulated Eqs. (2) to predict tumor progression under different treatments, including monotherapy and combination therapy. When DrugA was used as first-line treatment (**Figures 2A, D**), Type-W and Type-Y decreased, whereas Type-X and Type-Z increased. Once Type-X became the dominant population, the tumor started to grow again and would no longer be sensitive to the first treatment. We then changed this drug to DrugB. In this study, we set the drug-switching time at day 307 (*t*= 307) based on clinical observations of the median Progression-Free Survival (mPFS) of erlotinib treatment (21). Under the second-line medication of DrugB, the growth of Type-W and Type-X was suppressed, but that of Type-Y and Type-Z increased. Finally, Type-Z became the major population. The simulation results demonstrated a trend of tumor evolution under erlotinib-first treatment. At the end of our simulation period, set as day 1000 (*t*=1000), the cell number of the tumor was 2.12× 10^12^. Next, we examined the DrugB-first treatment. The main population changed from Type-W to Type-Y and Type-Z (**Figures 2B, E**). During the treatment period, Type-W and Type-X decreased, whereas Type-Y and Type-Z increased. Once Type-Y became the main population, the tumor started to grow again and was no longer sensitive to DrugB. Herein, we switched drugs at day 567 (*t*=567) because the mPFS was approximately 567 days under the osimertinib treatment (21). When DrugA was used as the second-line treatment, Type-Y was suppressed, and Type-Z continued to grow and dominated the tumor. Compared with the presumed evolution (**Figure 2E**), our model profitably reflected the tumor progression of osimertinib-first treatment. In this treatment schedule, the tumor recurred at day 490, which was longer than that of erlotinib-first therapy. Additionally, at the end of our simulation period (*t*=1000), the total cell number was 1.09× 10^12^, which was less than that of the DrugA-first treatment.

Furthermore, we investigated the outcomes of combination therapy (DrugC) by using DrugA and DrugB at the same time as first-line treatment (**Figures 2C, F**). When DrugC was applied as the first-line treatment, Type-W, Type-X, and Type-Y decreased, and only Type-Z continued to increase because it was resistant to both DrugA and DrugB. Type-W is the main population in the early phase of treatment, and eventually, Type-Z replaced Type-W to become the main population in the tumor. During medication, neither X nor Type-Y dominated the population. At day 1000, the total cell number was 4.2 × 10^12^.

### Drug-switching time and final cell number

**Figure 3:**
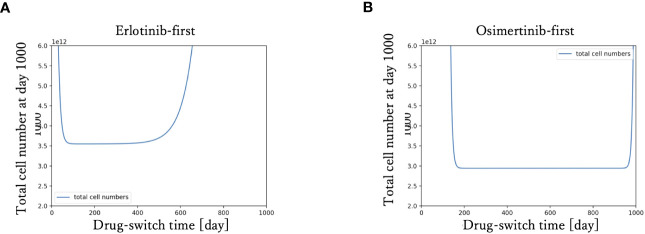
Drug switch time and final total cell number. The x-axis is drug-switch time, and the y-axis is the total cell number at day 1000. In panel **(A)**, the simulation result using erlotinib as a first-line treatment is shown. The lowest total cell number at day 1000 is approximately 2 × 10^12^. In panel **(B)**, the case of first-line Osimertinib treatment is shown. The lowest total cell number at day 1000 is approximately 1 × 10^12^.

To examine the relationship between drug-switching time and the development of the total cell number of the tumor, we simulated the tumor dynamics and measured the total cell number at day 1000 with various drug-switching times (**Figure 3**). In the case of DrugA-first treatment (**Figure 3A**), the lowest final total number of cells was 2.0× 10^12^, while it was about 1.0 × 10^12^ in the case of DrugB-first treatment (**Figure 3B**). This implied that first-line treatment with DrugB displayed better treatment outcomes than DrugA-first treatment. Moreover, the total number of cells at day 1000 remained essentially the same in an appropriate range of drug-switching times under both DrugA- and DrugB-first treatments. This suggested the existence of an optimal drug-switch period, and it was not advisable to switch drugs too early or too late. Furthermore, comparing the suitable drug-switching time period for these two treatments, DrugB-first therapy had a broader range than DrugA-first. In the DrugB-first treatment, switching drugs from days 200 to 900 was acceptable (**Figure 3B**). However, in DrugA-first therapy, the suitable drug-switching time ranged from day 100 to day 450 (**Figure 3A**).

### Cell initial proportion dependence

**Figure 4:**
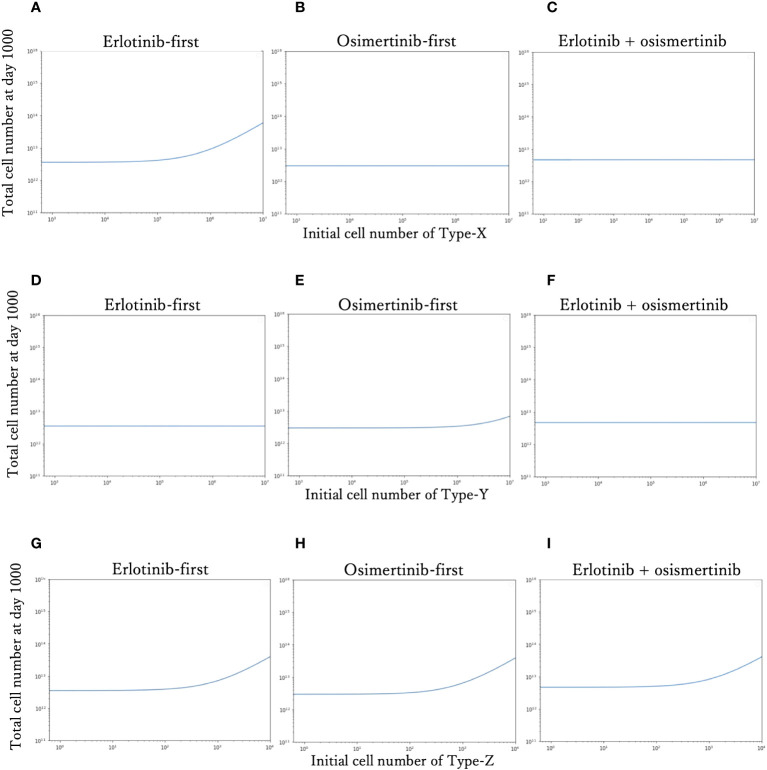
Relationship between the initial proportion and final cell number. The total cell number at day 1000 of each therapy with the different initial cell numbers of Type-X cells are shown in **(A–C)**. The final total cell number of each therapy with the different initial cell numbers of Type-Y cells are shown in **(D–F)**. The total cell number at day-1000 of each therapy with the different initial cell numbers of Type-Z cells are shown in **(G–I)**. The x-axis is initial proportion of mutation cells, the y-axis is the final total cell number.

To investigate the effect of the initial proportion of different mutant cells on the final cell number, we simulated how the final cell number changes with the increase of mutant cell proportion in different treatment strategies (**Figure 4**). We explored the effect of only one resistant cell type at one time, keeping other conditions constant as the standard condition. For Type-X and Type-Y, we tested the change in initial proportion from 10^-8^ to 10^-1^, and for Type-Z from 10^-9^ to 10^-5^. With the increase of Type-X cell (**Figures 4A–C**), the final cell number did not change under DrugB-first therapy (**Figure 4B**) and combination therapy (DrugC) (**Figure 4C**) but increased in DrugA-first therapy (**Figure 4A**) once the initial proportion of Type-X exceeded 10^-4^. Similarly, in Type-Y dependence simulations (**Figures 4D–F**), the final cell number increased only in DrugB-first therapy (**Figure 4E**) when the initial proportion of Type-Y became larger than 10^-3^. In addition, in Type-Z dependence simulations (**Figures 4G–I**), the final cell number increased once the initial proportion of Type-Z cell became larger than 10^-7^ under all treatments. Within the range of initial cell proportion that did not cause an increase in the final cell number, DrugB-first therapy always showed the smallest number of total cells at day 1000.

### Therapy selection map

**Figure 5:**
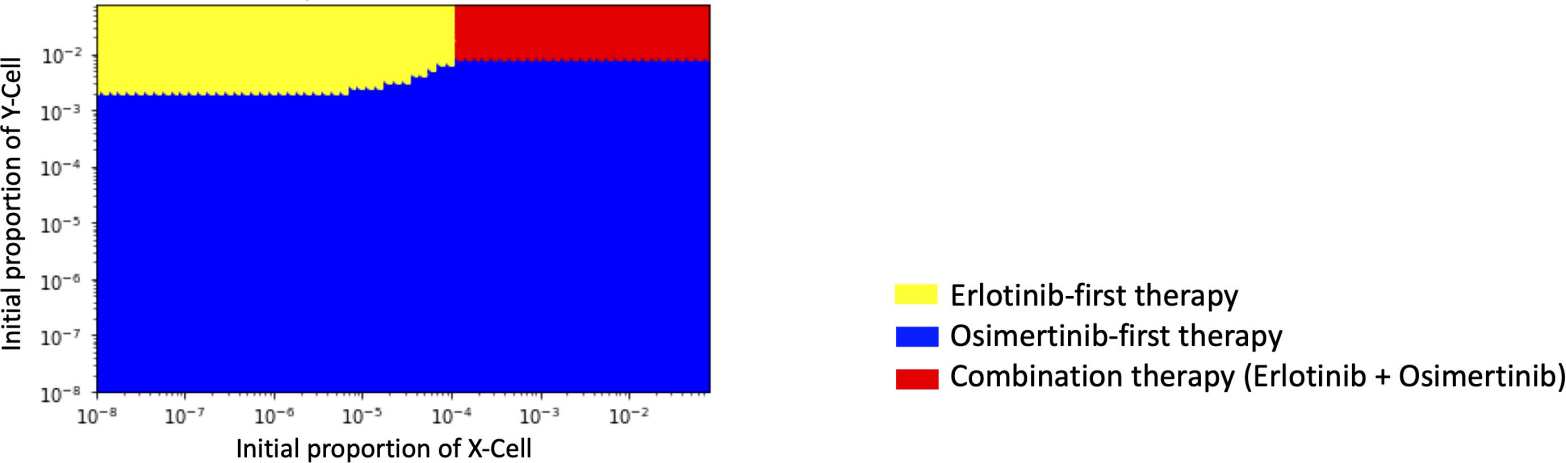
Initial Proportions of Type-X and -Y cells and treatment strategy selection. The x-axis is the initial ratio of Type-X, and the y-axis is the initial ratio of Type-Y. The yellow region means that osimertinib-first therapy is the optimal therapy, the blue region means erlotinib-first therapy is the optimal therapy, and the green region means combination therapy (erlotinib+osimertinib) is the optimal choice.

In order to identify which treatment strategy is optimal in a given case, we compared the final total cell number in different treatments with the change of the initial Type-X and Type-Y cell proportion and summarized the results in a therapy selection map (**Figure 5**). In this simulation, we kept the initial number of Type-Z constant as 10. By comparing the final total cell number under these three treatment strategies in the different initial proportions of Type-X and Type-Y cells, we determined the best strategy by realizing the smallest cell number at day 1000. The simulations were performed in the same method as used in **Figure 2**. From this map, we noticed that DrugB-first therapy was the optimal choice when tumors harbored a low initial proportion of Type-Y cells. However, DrugA-first therapy could still be advisable if the initial proportion of Type-Y cells was more significant in the tumor cluster. Furthermore, this map indicated that when both Type-X and Y cells had a high initial proportion in the tumor cluster, combination therapy (DrugC) was the optimal choice.

### Parameter sensitivity analysis

**Figure 6:**
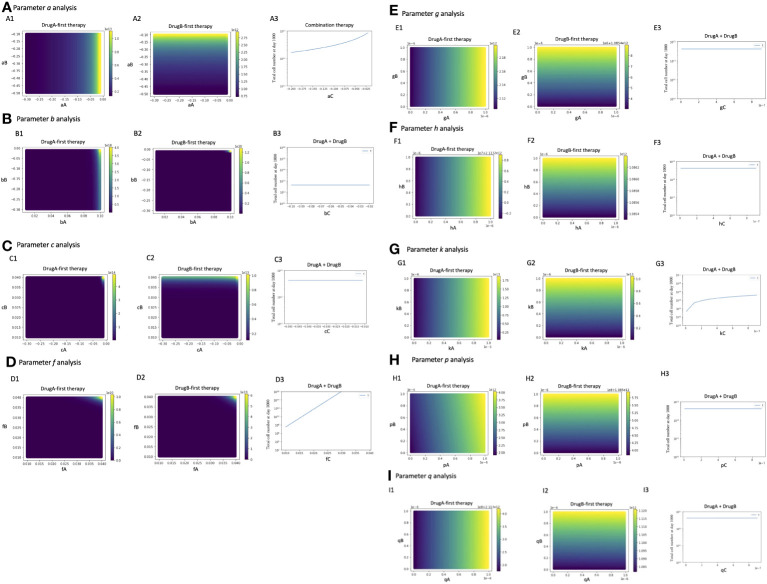
Parameter sensitivity analysis. Parameter dependence on the total total cell number at day 1000 under the three therapy strategies was analyzed. In monotherapy related analysis, the x- and y-axis are the parameters in the effect of DrugA and DrugB, and the color bar presented the final total cell number. In combination therapy, the x-axis is the parameter, while the y-axis is the final total cell number. The analysis of growth rates, *a*, *b*, *c*, *f* is showed in **(A–D)**, the mutation rates analysis is showed in **(E–I)**.

To investigate the parameter sensitivity, we analyzed how the total cell number at day 1000 changed with parameters under those three treatment strategies (**Figure 6**). In the analysis of the growth rate of Type-W cell (*a*), the final total cell number increased with the increase of *a_A_* under DrugA-first therapy (**Figure 6A1**); the increase of *a_B_* under DrugB-first therapy (**Figure 6A2**); and the increase of *a_C_* under the combination therapy (**Figure 6A3**). As for the growth rate of Type-X cell (*b*) and Type-Y cell (*c*), they did not affect the final total cell number significantly in our simulated value range (**Figures 6B, C**). Moreover, about the growth rate of Type-Z cell (*f)*, the final total cell number increased with *f_C_* under the combination therapy (DrugC) (**Figure 6D**). Concerning the effect of mutation rates (**Figure 6E–I**), their influence was different based on therapy strategies. In DrugA-first therapy, the increase of *g_A_*, *h_A_*, *k_A_*, *p_A_* and *q_A_* increased the final total cell number. Meanwhile, the increase of *g_B_*, *h_B_*, *k_B_*, *p_B_* and *q_B_* increased it in DrugB-first therapy. In the combination therapy (DrugC), only *k_C_* increased it (**Figure 6**).

### Parameter dependence on the therapy selection map

**Figure 7:**
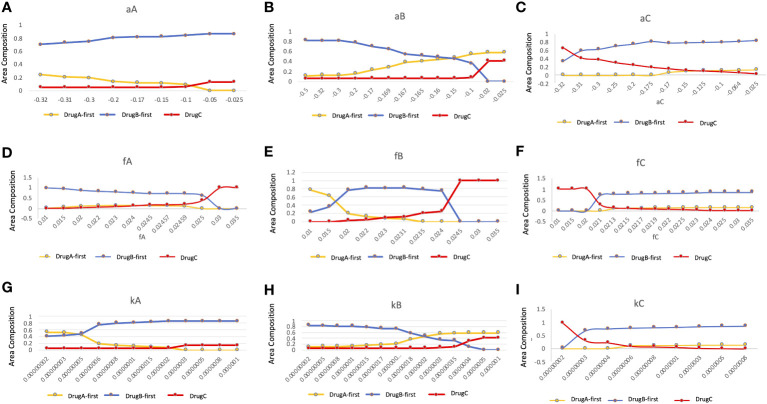
Parameter dependence on the area compositions of the three strategies in the optimal strategy map. We analyzed the composition of optimal therapies among DrugA-first therapy, DrugB-first therapy, and DrugC therapy in therapy selection map with the change of parameters. The x-axis is the parameter to be focused, the y-axis is the percentage of the area of the optimal therapies in the therapy selection map. The dependence of growth rates of Type-W **(A–C)**, and Type-Z cell **(D–F)**, and mutation rate from Type-W to Type-Z cell **(G–I)** under the three treatment strategies were tested. The yellow, blue, and red lines are DrugA-first therapy, DrugB-first therapy, and DrugC therapy, respectively.

Since several parameter values were set by our own assumptions, we investigated how these values affected the optimal choice of treatment in detail (**Figure 7**). In this analysis, we changed one focused parameter value, made a therapy-selection map as described in **Figure 5**, calculated the area of each strategy on the map and showed the area composition of each strategy at each parameter value. Especially, we investigated the dependence of growth rates of Type-W (*a*) and Type-Z (*f*) cell, and mutation rate from Type-W to Type-Z cell (*k*) under the three treatment strategies. As a result, the area where DrugB-first therapy exhibited superiority was large in the *a_A_*, *a_B_*, and *a_C_*,-dependent analyses (**Figures 7A–C**). When the DrugB effect was weak against Type-W cell, the DrugA-first therapy became superior (**Figure 7B**). Moreover, when we changed growth rates of Type-Z under the three strategies, the DrugB-first therapy was the best option again except the cases where the growth rate of Type-Z under DrugA and DrugB was large (**Figures 7D, E**), and the growth rate of Type-Z under DrugB and DrugC was small (**Figures 7E, F**). Finally, changing the mutation rate under the three treatment strategies, DrugB-first therapy was the best option in most cases (**Figures 7G–I**). When the mutation rate (*k*) was small under DrugA and DrugC, and large under DrugB, DrugA-first or DrugC therapy became the best option (**Figures 7G–I**).

## Discussion

In this study, we proposed a new mathematical model of EGFR-mutated NSCLC. First, our model successfully reproduced the process of tumor evolution under different treatment schedules, including monotherapy and combination therapy (**Figure 2**). In the erlotinib-first treatment (i.e., DrugA-first treatment), the drug was switched at day 307, while at day 576 it was switched in the osimertinib-first treatment (i.e., DrugB-first treatment). Next, we compared the effects of the two therapies. Our simulation results indicated that first-line osimertinib therapy was better than erlotinib. Within the same time period, for example, 1000 days in our study, osimertinib-first therapy resulted in a lower total cell number. Furthermore, the tumor recurred at nearly 500 days in osimertinib-first therapy compared to approximately 300 days in erlotinib-first therapy. This implied that first-line osimertinib therapy could suppress the growth of tumors more effectively than first-line erlotinib therapy, and could prolong the time of tumor recurrence. In the FLAURA project, clinical statistical data also revealed that EGFR-mutated NSCLC patients treated with osimertinib-first therapy had longer median mPFS (20, 21). This statistical study indicated the validity of our proposed model.Additionally, we noticed that in monotherapy, the total cell count was relatively low over a range of drug-switching times (**Figure 3**). This finding describes the existence of a suitable drug-switching phase, which suggests that it is not advisable to change the drug at a very early or late stage. In the suitable range of drug switching times, our simulation results showed that osimertinib-first therapy had a relatively lower total cell number than erlotinib-first therapy at day 1000. This result also indicates the potential of osimertinib as a first-line therapy in clinical applications. In addition, the appropriate drug-switching time range in osimertinib-first treatment was broader than that in erlotinib-first.

Furthermore, we explored the influence of tumor heterogeneity on therapeutic effects (**Figure 4**). By analyzing the relationship between the initial cell number and total cell number at the end of the tested time, we learned that the therapeutical effects depended on the initial ratio of resistant types in untreated tumors, and sensitive type did not affect it. According to the simulation results, when the initial ratio of Type-X exceeded the threshold, only the total cell number in the erlotinib-first therapy became large (**Figure 4A**). In the case of Type-Y, only osimertinib-first therapy resulted in large number of cells (**Figure 4E**). As for Type-Z, when its number became sufficiently large, the final total cell number developed rapidly in all the tested treatment schedules (**Figures 4G–I**). These results indicated that a high proportion of drug-resistant cells is associated with poor treatment efficacy. This conclusion suggests that if the tumor harbors a high ratio of Type-X, osimertinib-first is better than erlotinib-first. However, with a high initial ratio of Type-Y, erlotinib-first was better. Importantly, by combining this information, we for the first time theoretically revealed the relationship between the choice of treatment strategy and the initial proportion of Type-X and -Y cell (**Figure 5**). These findings indicated the advantage of first-line osimertinib treatment and revealed the influencing factors when determining treatment plans. Parameter sensitivity analysis about the total cell number and the best treatment choice confirmed the region where osimertinib-first therapy was superior to other options (**Figures 6** and **7**). Especially, we noticed that among the parameters, growth rate of Type-W and Type-Z cell and mutation rate from Type-W to Type-Z made a significantly change in the therapy selection map (**Figure 7**). Thess findings indicated the importance of suppressing all-drug-sensitive (Type-W) and all-drug-resistant (Type-Z) cells. This implied that during the treatment, not only the emergence of secondary resistant cells, but also the response of all-drug-resistant and -sensitive cells to drugs should be considered.

Furthermore, the simulation results showed that the combination of two types of EGFR-TKIs (erlotinib + osimertinib) as first-line therapy has the potential for clinical applications. Recently, several clinical studies have combined two types of EGFR-TKIs (first- and third-generation TKIs) as first-line treatment. In 2017, Wang et al. first reported the combination of erlotinib and osimertinib in patients with EGFR-mutated NSCLC patients (37). They illustrated the expediency of this type of treatment strategy. In addition, Rotow et al. applied gefitinib plus osimertinib as the first-line treatment for untreated patients with EGFR-mutated NSCLC (43). Their results showed the feasibility of conducting EGFR-TKI combination therapy, and survival analysis is in progress. In this study, we explored the combination of erlotinib and osimertinib as first-line therapy and explained the advantages of this method from a theoretical level. Especially, the drug response time with combination therapy was longer than that with monotherapy. Based on our simulation results, the recurrence time under combination therapy was longer than 500 days, whereas it was approximately 450 days in osimertinib-first treatment and 300 days in erlotinib-first therapy. This finding implied that the ability of combination therapy to prevent the emergence of acquired mutations and prolong the drug response time was even better than osimertinib-first treatment, which suggested its potential in clinical applications.

Based on the above, the versatility exhibited by the simulation results suggests that our model has the potential to be applied to simulate other similar cases in different cancer types. For further study, some clinical information about patients, such as age, sex, and the degree of malignancy of the tumor, may be considered in the parameter estimation. Thus, this model can be used to develop individual treatment schedules in the future.

## Data availability statement

The original contributions presented in the study are included in the article/Supplementary Material. Further inquiries can be directed to the corresponding author.

## Ethics statement

Ethical approval was not required for the study involving humans in accordance with the local legislation and institutional requirements. Written informed consent to participate in this study was not required from the participants or the participants’ legal guardians/next of kin in accordance with the national legislation and the institutional requirements.

## Author contributions

SSK and HH conceived the idea of the study. QY and HH developed simulation codes. QY conducted computational simulations. QY and HH contributed to the interpretation of the results. SSK and HH supervised the conduct of this study. All authors reviewed the manuscript draft and revised it. All authors contributed to the article and approved the submitted version.

## References

[1] Siegel RL Miller KD Jemal A. Cancer statistics, 2018. CA: A Cancer J Clin (2018) 68(1):7–30. doi: 10.3322/caac.21442 29313949

[2] Saito M Shiraishi K Kunitoh H Takenoshita S Yokota J Kohno T. Gene aberrations for precision medicine against lung adenocarcinoma. Cancer Sci (2016) 107(6):713–20. doi: 10.1111/cas.12941 PMC4968599 27027665

[3] Liu X Wang P Zhang C Ma Z. Epidermal growth factor receptor (EGFR): A rising star in the era of precision medicine of lung cancer. Oncotarget (2017) 8(30):50209–20. doi: 10.18632/oncotarget.16854 PMC5564844 28430586

[4] Rossi S D'Argento E Basso M Strippoli A Dadduzio V Cerchiaro E. Different EGFR gene mutations in exon 18, 19 and 21 as prognostic and predictive markers in NSCLC: A single institution analysis. Mol Diagn Ther (2016) 20(1):55–63. doi: 10.1007/s40291-015-0176-x 26645830

[5] Kobayashi Y Togashi Y Yatabe Y Mizuuchi H Jangchul P Kondo C. EGFR exon 18 mutations in lung cancer: molecular predictors of augmented sensitivity to afatinib or neratinib as compared with first- or third-generation TKIs. Clin Cancer Res (2015) 21(23):5305–13. doi: 10.1158/1078-0432.CCR-15-1046 26206867

[6] Castellano GM Aisner J Burley SK Vallat B Yu HA Pine SR. A novel acquired exon 20 EGFR M766Q mutation in lung adenocarcinoma mediates osimertinib resistance but is sensitive to neratinib and poziotinib. J Thorac Oncol (2019) 14(11):1982–8. doi: 10.1016/j.jtho.2019.06.015 PMC8080261 31254668

[7] Yasuda H Park E Yun CH Sng NJ Lucena-Araujo AR Yeo WL. Structural, biochemical, and clinical characterization of epidermal growth factor receptor (EGFR) exon 20 insertion mutations in lung cancer. Sci Transl Med (2013) 5(216):216ra177. doi: 10.1126/scitranslmed.3007205 PMC3954775 24353160

[8] Castellanos E Feld E Horn L. Driven by mutations: the predictive value of mutation subtype in EGFR-mutated non-small cell lung cancer. J Thorac Oncol (2017) 12(4):612–23. doi: 10.1016/j.jtho.2016.12.014 28017789

[9] O'Kane GM Bradbury PA Feld R Leighl NB Liu G Pisters KM. Uncommon EGFR mutations in advanced non-small cell lung cancer. Lung Cancer (2017) 109:137–44. doi: 10.1016/j.lungcan.2017.04.016 28577943

[10] Costa DB. Kinase inhibitor-responsive genotypes in EGFR mutated lung adenocarcinomas: moving past common point mutations or indels into uncommon kinase domain duplications and rearrangements. Transl Lung Cancer Res (2016) 5(3):331–7. doi: 10.21037/tlcr.2016.06.04 PMC4931120 27413714

[11] Ward WH Cook PN Slater AM Davies DH Holdgate GA Green LR. Epidermal growth factor receptor tyrosine kinase. Investigation of catalytic mechanism, structure-based searching and discovery of a potent inhibitor. Biochem Pharmacol (1994) 48(4):659–66. doi: 10.1016/0006-2952(94)90042-6 8080438

[12] Pao W Miller V Zakowski M Doherty J Politi K Sarkaria I. EGF receptor gene mutations are common in lung cancers from "never smokers" and are associated with sensitivity of tumors to gefitinib and erlotinib. Proc Natl Acad Sci U.S.A. (2004) 101(36):13306–11. doi: 10.1073/pnas.0405220101 PMC516528 15329413

[13] Rotow J Bivona TG. Understanding and targeting resistance mechanisms in NSCLC. Nat Rev Cancer (2017) 17(11):637–58. doi: 10.1038/nrc.2017.84 29068003

[14] Park K Tan EH O'Byrne K Zhang L Boyer M Mok T. Afatinib versus gefitinib as first-line treatment of patients with EGFR mutation-positive non-small-cell lung cancer (LUX-Lung 7): a phase 2B, open-label, randomised controlled trial. Lancet Oncol (2016) 17(5):577–89. doi: 10.1016/S1470-2045(16)30033-X 27083334

[15] Park K Wan-Teck Lim D Okamoto I Yang JC. First-line afatinib for the treatment of EGFR mutation-positive non-small-cell lung cancer in the 'real-world' clinical setting. Ther Adv Med Oncol (2019) 11:1758835919836374. doi: 10.1177/1758835919836374 31019567 PMC6466470

[16] Wu YL Zhou C Liam CK Wu G Liu X Zhong Z. First-line erlotinib versus gemcitabine/cisplatin in patients with advanced EGFR mutation-positive non-small-cell lung cancer: analyses from the phase III, randomized, open-label, ENSURE study. Ann Oncol (2015) 26(9):1883–9. doi: 10.1093/annonc/mdv270 26105600

[17] Kobayashi S Ji H Yuza Y Meyerson M Wong KK Tenen DG. An alternative inhibitor overcomes resistance caused by a mutation of the epidermal growth factor receptor. Cancer Res (2005) 65(16):7096–101. doi: 10.1158/0008-5472.CAN-05-1346 16103058

[18] Yun CH Mengwasser KE Toms AV Woo MS Greulich H Wong KK. The T790M mutation in EGFR kinase causes drug resistance by increasing the affinity for ATP. Proc Natl Acad Sci U.S.A. (2008) 105(6):2070–5. doi: 10.1073/pnas.0709662105 PMC2538882 18227510

[19] Soejima K Yasuda H Hirano T. Osimertinib for EGFR T790M mutation-positive non-small cell lung cancer. Expert Rev Clin Pharmacol (2017) 10(1):31–8. doi: 10.1080/17512433.2017.1265446 27885838

[20] Soria JC Ohe Y Vansteenkiste J Reungwetwattana T Chewaskulyong B Lee KH. FLAURA investigators. Osimertinib in untreated EGFR-mutated advanced non-small-cell lung cancer. N Engl J Med (2018) 378(2):113–25. doi: 10.1056/NEJMoa1713137 29151359

[21] Ramalingam SS Vansteenkiste J Planchard D Cho BC Gray JE Ohe Y. FLAURA investigators. Overall survival with osimertinib in untreated, EGFR-mutated advanced NSCLC. N Engl J Med (2020) 382(1):41–50. doi: 10.1056/NEJMoa1913662 31751012

[22] Cross DA Ashton SE Ghiorghiu S Eberlein C Nebhan CA Spitzler PJ. overcomes T790M-mediated resistance to EGFR inhibitors in lung cancer. Cancer Discovery (2014) 4(9):1046–61. doi: 10.1158/2159-8290.CD-14-0337 PMC4315625 24893891

[23] Leonetti A Sharma S Minari R Perego P Giovannetti E Tiseo M. Resistance mechanisms to osimertinib in EGFR-mutated non-small cell lung cancer. Br J Cancer (2019) 121(9):725–37. doi: 10.1038/s41416-019-0573-8 PMC6889286 31564718

[24] Oxnard GR Hu Y Mileham KF Husain H Costa DB Tracy P. Assessment of resistance mechanisms and clinical implications in patients with EGFR T790M-positive lung cancer and acquired resistance to osimertinib. JAMA Oncol (2018) 4(11):1527–34. doi: 10.1001/jamaoncol.2018.2969 PMC6240476 30073261

[25] Wang S Tsui ST Liu C Song Y Liu D. EGFR C797S mutation mediates resistance to third-generation inhibitors in T790M-positive non-small cell lung cancer. J Hematol Oncol (2016) 9(1):59. doi: 10.1186/s13045-016-0290-1 27448564 PMC4957905

[26] Mok TS Wu YL Thongprasert S Yang CH Chu DT Saijo N. Gefitinib or carboplatin-paclitaxel in pulmonary adenocarcinoma. N Engl J Med (2009) 361(10):947–57. doi: 10.1056/NEJMoa0810699 19692680

[27] Rosell R Carcereny E Gervais R Vergnenegre A Massuti B Felip E. Erlotinib versus standard chemotherapy as first-line treatment for European patients with advanced EGFR mutation-positive non-small-cell lung cancer (EURTAC): a multicentre, open-label, randomised phase 3 trial. Lancet Oncol (2012) 13(3):239–46. doi: 10.1016/S1470-2045(11)70393-X 22285168

[28] Sequist LV Yang JC Yamamoto N O'Byrne K Hirsh V Mok T. Phase III study of afatinib or cisplatin plus pemetrexed in patients with metastatic lung adenocarcinoma with EGFR mutations. J Clin Oncol (2013) 31(27):3327–34. doi: 10.1200/JCO.2012.44.2806 23816960

[29] Michor F Hughes TP Iwasa Y Branford S Shah NP Sawyers CL. Dynamics of chronic myeloid leukaemia. Nature (2005) 435(7046):1267–70. doi: 10.1038/nature03669 15988530

[30] Diaz LA Jr Williams RT Wu J Kinde I Hecht JR Berlin J. The molecular evolution of acquired resistance to targeted EGFR blockade in colorectal cancers. Nature (2012) 486(7404):537–40. doi: 10.1038/nature11219 PMC3436069 22722843

[31] Foo J Michor F. Evolution of acquired resistance to anti-cancer therapy. J Theor Biol (2014) 355:10–20. doi: 10.1016/j.jtbi.2014.02.025 24681298 PMC4058397

[32] Turajlic S Sottoriva A Graham T Swanton C. Resolving genetic heterogeneity in cancer. Nat Rev Genet (2019) 20(7):404–16. doi: 10.1038/s41576-019-0114-6 30918367

[33] Altrock PM Liu LL Michor F. The mathematics of cancer: integrating quantitative models. Nat Rev Cancer (2015) 15(12):730–45. doi: 10.1038/nrc4029 26597528

[34] Castagnino N Maffei M Tortolina L Zoppoli G Piras D Nencioni A. Systems medicine in colorectal cancer: from a mathematical model toward a new type of clinical trial. Wiley Interdiscip Rev Syst Biol Med (2016) 8(4):314–36. doi: 10.1002/wsbm.1342 PMC6680205 27240214

[35] Jamal-Hanjani M Wilson GA McGranahan N Birkbak NJ Watkins TBK Veeriah S. Tracking the evolution of non-small-cell lung cancer. N Engl J Med (2017) 376(22):2109–21. doi: 10.1056/NEJMoa1616288 28445112

[36] Gridelli C Rossi A Carbone DP Guarize J Karachaliou N Mok T. Non-small-cell lung cancer. Nat Rev Dis Primers (2015) 1:15009. doi: 10.1038/nrdp.2015.9 27188576

[37] Wang Z Yang JJ Huang J Ye JY Zhang XC Tu HY. Lung adenocarcinoma harboring EGFR T790M and in trans C797S responds to combination therapy of first- and third-generation EGFR TKIs and shifts allelic configuration at resistance. J Thorac Oncol (2017) 12(11):1723–7. doi: 10.1016/j.jtho.2017.06.017 28662863

[38] Oda N Hotta K Ninomiya K Minami D Ichihara E Murakami T. A phase II trial of EGFR-TKI readministration with afatinib in advanced non-small-cell lung cancer harboring a sensitive non-T790M EGFR mutation: Okayama Lung Cancer Study Group trial 1403. Cancer Chemother Pharmacol (2018) 82(6):1031–8. doi: 10.1007/s00280-018-3694-5 30276451

[39] Nakagawa K Garon EB Seto T Nishio M Ponce Aix S Paz-Ares L. RELAY Study Investigators. Ramucirumab plus erlotinib in patients with untreated, EGFR-mutated, advanced non-small-cell lung cancer (RELAY): a randomised, double-blind, placebo-controlled, phase 3 trial. Lancet Oncol (2019) 20(12):1655–69. doi: 10.1016/S1470-2045(19)30634-5 31591063

[40] Kohsaka S Nagano M Ueno T Suehara Y Hayashi T Shimada N. A method of high-throughput functional evaluation of EGFR gene variants of unknown significance in cancer. Sci Transl Med (2017) 9(416):eaan6566. doi: 10.1126/scitranslmed.aan6566 29141884

[41] Starrett JH Guernet AA Cuomo ME Poels KE van Alderwerelt van Rosenburgh IK Nagelberg A. Drug sensitivity and allele specificity of first-line osimertinib resistance EGFR mutations. Cancer Res (2020) 80(10):2017–30. doi: 10.1158/0008-5472.CAN-19-3819 PMC7392201 32193290

[42] Gunnarsson EB De S Leder K Foo J. Understanding the role of phenotypic switching in cancer drug resistance. J Theor Biol (2020) 490:110162. doi: 10.1016/j.jtbi.2020.110162 31953135 PMC7785289

[43] Rotow JK Botelho Costa D Paweletz CP Awad MM Marcoux P Rangachari D. Concurrent osimertinib plus gefitinib for first-line treatment of EGFR-mutated non-small cell lung cancer (NSCLC). J Clin Oncol (2020) 38(15_suppl):9507–7. doi: 10.1200/JCO.2020.38.15_suppl.9507

